# Prenatal and postnatal antibiotic exposure influences the gut microbiota of preterm infants in neonatal intensive care units

**DOI:** 10.1186/s12941-018-0264-y

**Published:** 2018-03-19

**Authors:** Zhi-Hui Zou, Dong Liu, Hong-Dong Li, Dan-Ping Zhu, Yu He, Ting Hou, Jia-Lin Yu

**Affiliations:** 1 Department of Neonatology, Children’s Hospital of Chongqing Medical University, Chongqing, 400014 China; 2 Department of Neonatology, Sichuan Maternal and Child Health Service Hospital, Chengdu, 610041 Sichuan China

**Keywords:** Infant, premature, Gastrointestinal microbiome, Anti-bacterial agents, High-throughput nucleotide sequencing, Intensive care unit, neonatal

## Abstract

**Background:**

To explore the influences of prenatal antibiotic exposure, the intensity of prenatal and postnatal antibiotic exposure on gut microbiota of preterm infants and whether gut microbiota and drug resistant strains in the neonatal intensive care unit (NICU) over a defined period are related.

**Methods:**

Among 28 preterm infants, there were two groups, the PAT (prenatal antibiotic therapy) group (12 cases), and the PAF (prenatal antibiotic free) group (12 cases). Fecal samples from both groups were collected on days 7 and 14. According to the time of prenatal and postnatal antibiotic exposure, cases were divided into two groups, H (high) group (11 cases) and L (low) group (11 cases), and fecal samples on day 14 were collected. Genomic DNA was extracted from the fecal samples and was subjected to high throughput 16S rRNA amplicon sequencing. Bioinformatics methods were used to analyze the sequencing results.

**Results:**

Prenatal and postnatal antibiotic exposure exercised influence on the early establishment of intestinal microflora of preterm infants. *Bacteroidetes* decreased significantly in the PAT group (*p* < 0.05). The number of *Bifidobacterium* significantly decreased in the PAT group and H group (*p *< 0.05). The early gut microbiota of preterm infants with prenatal and postnatal antibiotic exposure was similar to resistant bacteria in NICU during the same period.

**Conclusion:**

Prenatal and postnatal antibiotic exposure may affect the composition of early gut microbiota in preterm infants. Antibiotic-resistant bacteria in NICU may play a role in reshaping the early gut microbiota of preterm infants with prenatal and postnatal antibiotic exposure.

**Electronic supplementary material:**

The online version of this article (10.1186/s12941-018-0264-y) contains supplementary material, which is available to authorized users.

## Background

The human gastrointestinal tract contains diverse, complex, and dynamic communities of microorganisms. Most of these microorganisms are bacteria, the density of which is approximately 10^13^–10^14^ cells/g fecal matter. Gut microbiota contributes significantly to varied aspects of host health, including immunity, development, and behavior, while major shifts in complex microbial systems are associated with disease [1, 2]. Indeed, epidemiology and laboratory studies have found that gut microbiota imbalances are associated with several infectious diseases, chronic inflammatory bowel diseases, allergic diseases, autoimmune diseases, and some metabolic diseases [3–5].

Prenatal and postnatal periods are critical for the establishment of infant gut microbiota, which is susceptible to maternal factors, delivery mode, and feeding mode [6, 7]. The delay or disorder of preterm infant microbiota has a long-term effect on the physiology of the infant [8, 9]. Various environmental factors within NICU influence infant microbiota [10, 11]. The routine application of antibiotics in China is very popular, especially in NICU. Due to the high density of inpatients, nosocomial infections caused by resistant bacteria are frequent. However, the effects of antibiotic exposure on the gut microbiota of preterm infants in prenatal and postnatal periods remain undefined.

In this study, high-throughput amplicon sequencing was used to investigate the effects of prenatal antibiotic exposure and its intensity on the development of gut microbiota in preterm infants within 2 weeks of birth. We also explored whether there are similarities between the early gut microbiota and resistant bacteria colonized in the NICU.

## Methods

### Study population and design

This study was approved by the Ethics Committee of Bo’ai Hospital, Zhongshan, Guangdong Province. Twenty-eight preterm infants were enrolled in the study and the inclusion criteria were as follows: All the pregnant women were admitted to Bo’ai Hospital of Zhongshan before delivery, and the preterm infants were immediately hospitalized in the NICU of Bo’ai Hospital after delivery. Exclusion criteria were as follows: clinical use of probiotics, the presence of asphyxia, congenital malformations, congenital abnormalities, genetic metabolic diseases, or the presence of surgical diseases. For this study, two grouping strategies were established. Firstly, according to whether the infants were exposed to prenatal antibiotic therapy, 28 cases were divided into two groups: PAT (prenatal antibiotic therapy) group and PAF (prenatal antibiotic free) control group; each group had 12 patients. The other four patients were excluded because of difficulty in pairing. Secondly, according to antibiotic exposure intensity, i.e., total time of antibiotic exposure both before and after delivery, there were 11 pairs in two groups, i.e., > 7d group (H group) and ≤ 7d group (L group). These subjects were selected from the same 28 patients. For details on grouping, please refer to Additional files 1: Tables S1, S2 in Additional Materials. Cases and controls were matched by delivery method, gestational age, and feeding pattern. Fecal samples on days 7 (d7) and 14 (d14) were collected.

The antibiotic resistant strains present in Bo’ai Hospital, located in Guangdong, Southern China, were identified. According to the monthly report of drug resistant strains in the NICU, provided by the Infection Control Department of Bo’ai Hospital, the resistant bacteria colonized in the NICU were *Klebsiella pneumoniae* (40%), *Escherichia coli* (28.57%), and *Acinetobacter baumanii* (20%). The main prenatal antibiotics were cefazolin sodium pentahydrate, while the main antibiotics used in the NICU were cefuroxime sodium, cefoperazone-sulbactam, imipenem-cilastatin sodium, and meropenem. The prenatal antibiotic applied to the subjects in this study was exclusively cefazolin sodium pentahydrate, while the postnatal antibiotic was exclusively cefuroxime sodium.

### High throughput 16S rRNA amplicon sequencing

Fecal samples were collected with disposable sterile fecal collection tubes from soiled patient diapers, and delivered to the laboratory in an ice box. Then, 250 mg of each sample was put into sterile EP tubes. The samples of d7 and d14 of every infant were put into two to four EP tubes. All samples were stored in the refrigerator at − 80 °C. Genomic DNA was extracted from fecal samples using QIAamp FAST DNA Stool Mini Kit (Qiagen, Hilden, Germany), according to the manufacturer’s instructions.

The bacterial 16S rRNA gene amplicons (V3 + V4 regions) were generated with the following polymerase chain reaction (PCR) primers: Forward, 338F (5′-ACTCCTACGGGAGGCAGCA-3′); reverse, 806R (5′-GGACTACHVGGGTWTCTAAT-3′) [12]. PCRs were completed under the following conditions: 95 °C for 3 min, followed by 27 cycles of 95 °C for 30 s, 55 °C for 30 s, and 72 °C for 45 s, and finally, 72 °C for 10 min. PCR products were analyzed by agarose gel electrophoresis (2% in 0.25 × Tris–acetate-EDTA buffer), and were purified using the AxyPrep kit (AXYGEN, USA), according to the manufacturer’s protocol. The DNA amplicons were used to construct the sequencing libraries for the high throughput sequencing on an Illumina MiSeq platform.

### Bioinformatic analysis

In order to ensure bioinformatic analysis reliability, the raw sequencing reads were filtered before the biological analysis to assess the quality of the sequence. The specific criteria were as follows: (1) Remove sequences of less than 50 bp; (2) according to the overlap relationship, the sequences were spliced, and the minimum overlap length was 10 bp; (3) the maximum mismatch rate of the splicing sequence overlap region should not be higher than 0.2; (4) the base number of primer mismatches must be less than 2, and the barcode was not allowed to be mismatched.

After the sequences were optimized, operational taxonomic units (OTU) assignment was performed for all sequences at a 97% similarity level. OTU cluster analysis, taxonomy analysis, and species diversity analysis were performed. According to the results of the taxonomy analysis, the differences in the composition of the community structure were analyzed at the phylum and genus levels. The Shannon index and Simpson index were calculated to characterize the community diversity. Rarefaction curves, reflecting the sequencing depth, were calculated using custom R scripts. To characterize the richness in a specific gut community, the custom R scripts were used to obtain the Shannon–Wiener curve, Venn diagrams, and the microbial community bar plots. Principal coordinate analyses (PCoA), using the weighted and unweighted UniFrac distances, were calculated using the PCoA function of the R package Ape [13]. The gut microbial communities of different conditions were further compared using LDA Effect Size (LEfSe; http://huttenhower.sph.harvard.edu/galaxy). The differential features were identified on the OTU level.

## Results

### Effect of prenatal antibiotic exposure on the development of gut microbiota in preterm infants

In this study, there were no significant differences between the PAT and PAF groups in terms of the gestational age, birth weight, delivery mode, and feeding method (*p *> 0.05; Table 1). Statistically significant differences in gender were observed, and more boys were in the PAT group (*p *= 0.009). The detailed clinical data are shown in Table 1.

**Table 1:** Clinical data of the PAT and PAF groups

Clinical data	PAT infants	PAF infants	Statistical method	*P* value
Gestational age (d)	227.6 (mean) ± 7.6 (SD)	229.1 ± 7.3	Student’s t test	0.09
Birth weight (g)	1777.08 ± 297.1	1814.16 ± 169.14	Student’s t test	0.711
Delivery mode (CS/VD)	3/9	3/9	Chi square test	1
Gender (male/female)	11/1	8/4	Chi square test	0.009
Feeding (premature infant formula/mixed feeding/breastfeeding)	4/3/5	4/3/5	Chi square test	1
Postnatal antibiotic (d)	7 (ranging 3–13)	7 (ranging 3–14)	Non-parametric test	0.671
PROM	12/0	6/6	Chi square test	NA
Histological chorioamnionitis (positive/negative/no. of inspection)	3/6/3	2/6/4	Chi square test	NA

*VD* vaginally delivered, *CS* cesarean section, *PROM* premature rupture of membranes

**Fig. 1:**
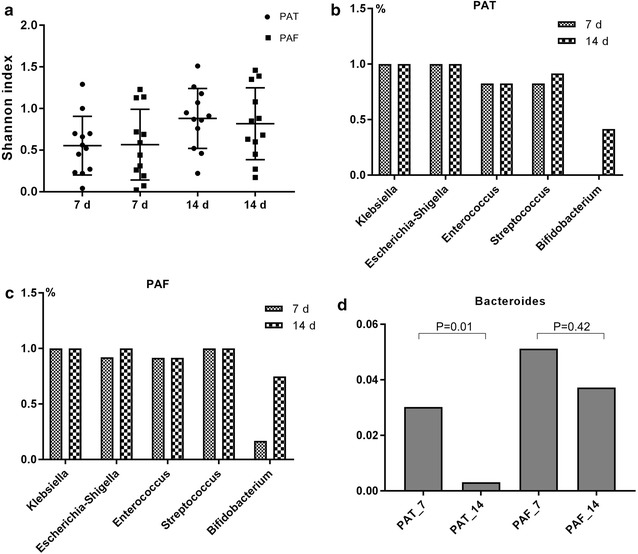
**a** Shannon index of PAT and PAF groups on postnatal d7 and d14. **b** Colonization percentage of five gut bacteria in the PAT group. **c** Colonization percentage of five gut bacteria in the PAF control group. **d** *Bacteriodetes* in the PAT and PAF groups on d7 and d14

The average sequence number of all samples was 448.21 and the sequencing depth reached about 99.5%. The rarefaction curves of different individuals were distinct (Additional file 1: Figure S1), indicating that there were significant inter-individual variations in gut bacterial diversity. The Shannon index of the PAT group was higher on d14 (0.88) than on d7 (0.55), so was that of the PAF group (0.57 on d7, 0.82 on d14). As time went by, there was an increasing trend in gut bacterial diversity. No significant differences were observed between the PAT and PAF groups on both d7 and d14 (Fig. 1a), which indicates the trivial influence of prenatal antibiotic exposure on gut microflora.

The number of OTUs on d7 and d14 in the PAT group was 31 and 81, respectively (Additional file1: Figure S2), with corresponding numbers in the PAF group of 31 and 79, respectively. In the PAT group, the number of shared OTUs between subjects on d7 and d14 was 27, while in the PAF group, it was 26. In both groups, there were more shared OTUs on d7 than on d14, indicating greater diversity in both groups on d14 than on d7 (*p* < 0.05).

At the phylum level on d7, *Proteobacteria* (PAF 79.57%, PAT 92.35%) and *Firmicutes* (PAF 9.73%, PAT 4.69%) were predominant in gut microbiota, followed by *Actinobacteria* (PAF 1.58%, PAT 0.02%) and *Bacteroidetes* (PAF 9.11%, PAT 2.93%). On d14, *Proteobacteria* (PAF 74.78%, PAT 87.22%) and *Firmicutes* (PAF 11.19%, PAT 10.61%) were predominant in gut microbiota, followed by *Actinobacteria* (PAF 8.21%, PAT 1.75%) and *Bacteroidetes* (PAF 5.75%, PAT 0.38%). The relative abundance of *Proteobacteria* was higher in the antibiotic exposure group than in the non-exposure group, on both days, while *Firmicutes* was less abundant in the former group than in the latter one, especially on d7. *Bacteroidetes* in both the PAF and PAT groups were lower on d14 than on d7, with a more marked decrease observed in the PAT group, with a statistically significant difference (*p* < 0.05), further highlighting the prolonged effect of prenatal antibiotic exposure on the establishment and development of neonatal gut microflora.

**Fig. 2:**
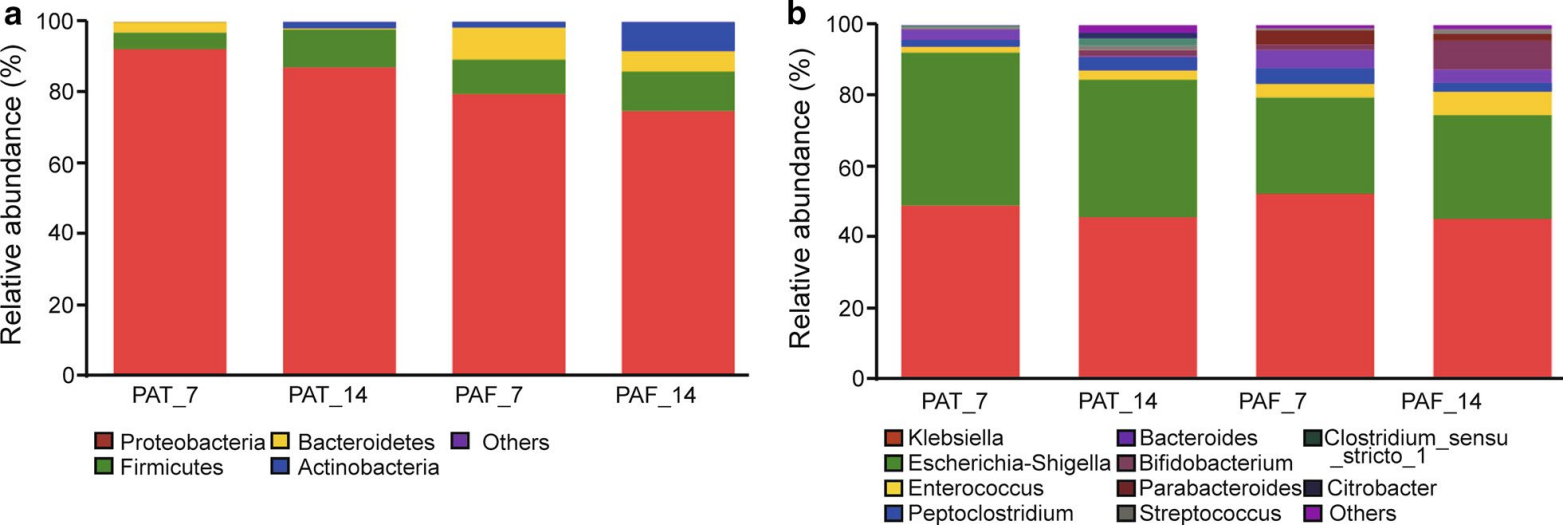
Relative abundance of the main gut bacterial groups. **a** At the phylum level. **b** At the genus level. On d7 and d14, gut microbiota were generally dominated by the phyla Proteobacteria and Firmicutes, followed by Actinobacteria and Bacteroidetes. At the genus level, *Klebsiella* and *Escherichia*-*Shigella* were dominant. On d14, bacteria such as *Bacteroides, Staphylococcus, Clostridium_sensu_stricto_1, Parabacteroides*, and *Bifidobacterium* were more abundant than on d7

At the genus level, *Klebsiella* (on d7, PAF 52.17%, PAT 48.96%; on d14, PAF 45.03%, PAT 45.81%) and *Escherichia*-*Shigella* (on d7, PAF 27.35%, PAT 43.35%; on d14, PAF 29.47%, PAT 40.36%) were predominant. Moreover, in the PAT group, the colonization of *Bifidobacterium* was delayed (0/12 vs. 3/12 on d7; 5/12 vs. 9/12 on d14). By d14, the relative abundance of some bacteria had increased substantially in the gut, such as *Bacteroides*, *Staphylococcus*, *Clostridium_sensu_stricto_1*, *Parabacteroides*, and *Bifidobacterium* (Fig. 2b). Moreover, in the PAT group, the colonization of *Bifidobacterium* was delayed (Fig. 1b). On d7 there was no colonization of *Bifidobacterium*, and by d14 the number of preterm infants colonized by *Bifidobacterium* was significantly lower in the PAT group than in the PAF group (0/12 vs. 3/12 on d7; 5/12 vs. 9/12 on d14; *p* < 0.05; Fig. 1b and c). *Bacteroidetes* decreased in both groups with increasing antibiotic exposure time, and this decrease was more significant in the PAT group than the PAF group (*p* < 0.05; Fig. 1d).

The predominant bacteria of PAT and PAF groups were similar to the drug resistant strains in NICU of the same period.

**Fig. 3:**
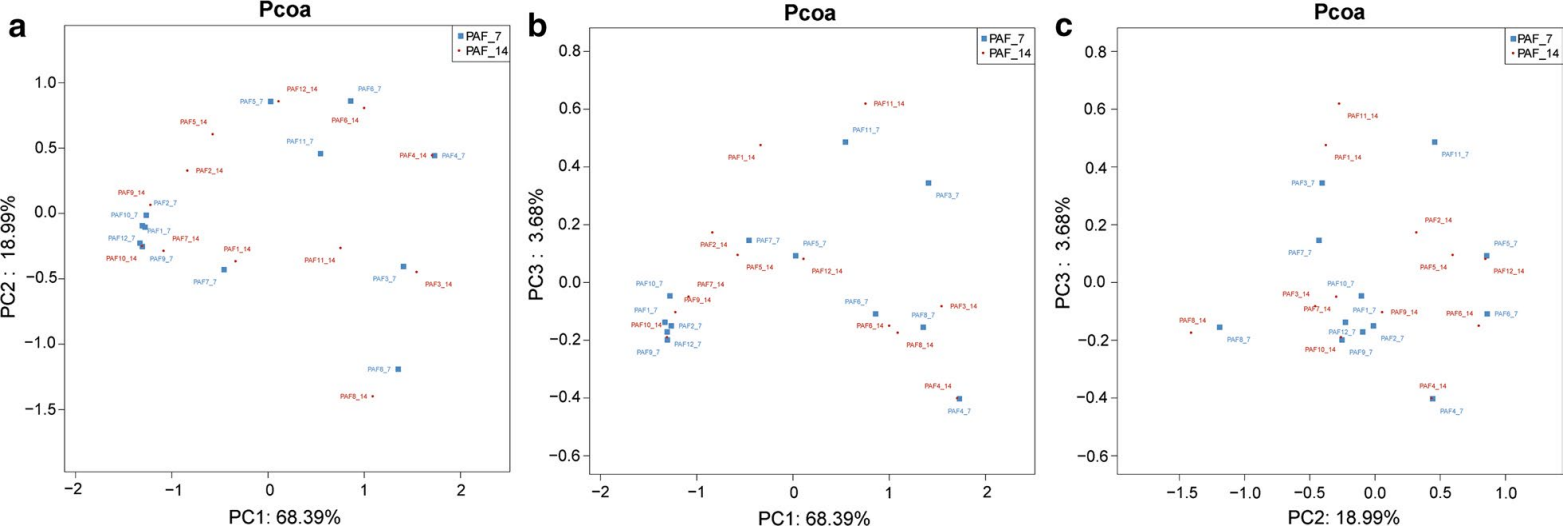
Comparison of microbial communities by PCoA. PCoA was generated with OTUs (at 97% similarity) present in the different fecal samples. **a** PC1 vs. PC2. **b** PC1 vs. PC3. **c** PC2 vs. PC3

The extent of variation within the gut bacteria population in the different samples correlated with the human host, infant growth and development, prenatal/postnatal antibiotic exposure, and so on. In PCoA (Fig. 3), each spot of the PAT and PAF group members were separated well, but neither those of the PAT group nor those of the PAF group clustered together, indicating that antibiotic exposure did not determine the gut microbiota development. As shown in Fig. 3, dots from both datasets were dispersed and intermingled, suggesting that the bacteria community composition of PAF_7 was largely similar to that of PAF_14.

LEfSe is a method for metagenomic biomarker discovery by way of class comparison, tests of biological consistency, and effect size estimation [14]. The differential features were identified on the OTU level. The LEfSe analysis of d7 showed that PAT and PAF groups were not significantly different.

**Fig. 4:**
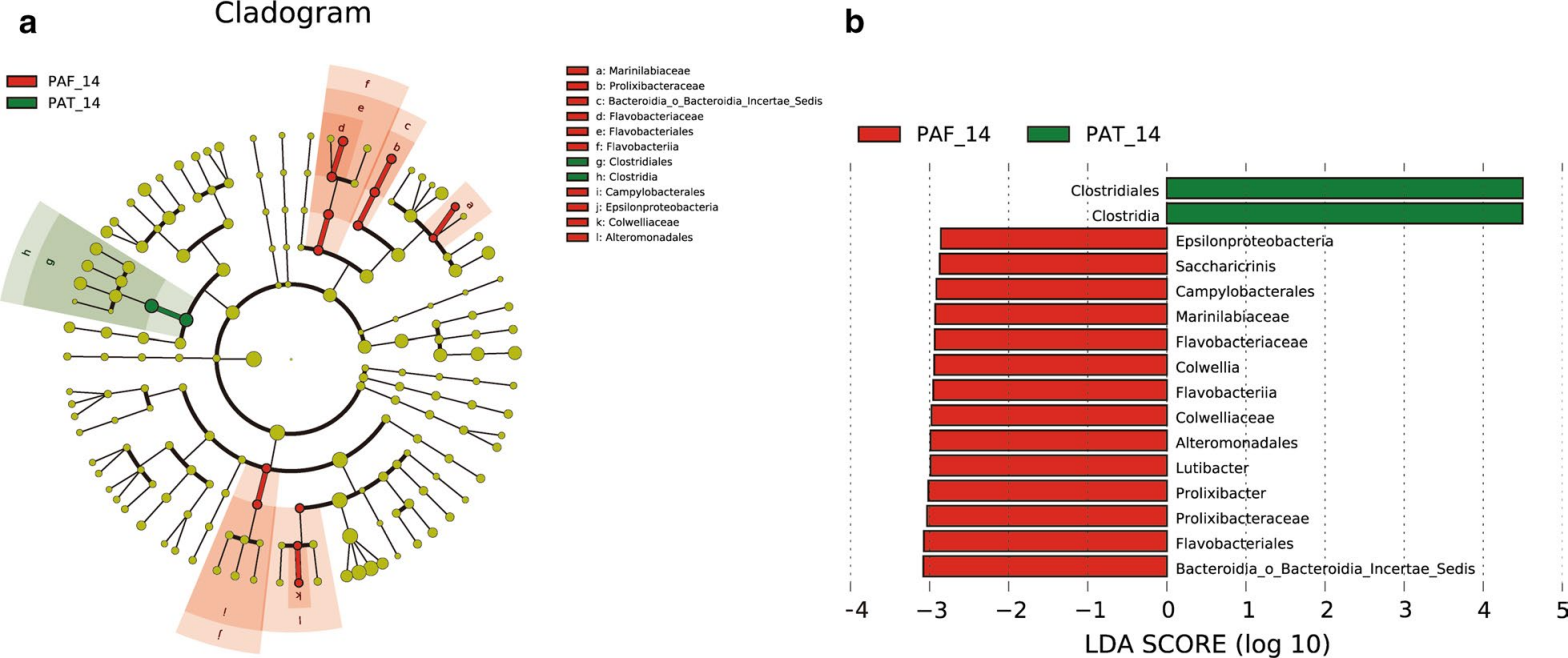
LEfSe results on gut microbiomes of preterm infants. **a** Clustering tree. Different color areas represent different groups. The yellow nodes represent microbial taxa without significant differences between PAT and PAF groups. The red nodes represent microbial taxa that played an important role in the PAF group, and green nodes represent microbial taxa that played an important role in the PAT group. **b** Histogram showing the distribution of LDA values

LEfSe analysis of d14 finds the relative abundance of *Saccharicrinis*, *Acteroidia, Epsilonproteobacteria*, *Incertae_Sedis*, and *Flavobacteriales* was significantly higher in the PAT group than in the PAF control group, and the relative abundance of *Clostridiales* and *Clostridia* was much lower in the former than in the latter (Fig. 4a and b).

### Effects of antibiotic exposure intensity on the development of intestinal microbiota in preterm infants

The preterm infants were divided into two groups according to the antibiotic treatment time: > 7d group (H group) and ≤ 7d group (L group). Delivery modes, gestational age, feeding pattern, and other factors that may affect the intestinal microbiota, were matched between the two groups (Table 2). Twenty-two preterm infants were enrolled. The fecal samples on d14 were collected.

**Table 2:** Clinical data of prenatal antibiotic treatment H group and L group

Clinical data	H group	L group	Statistical method	*P* value
Gestational age (d)	227 (ranging 218–237)	230 (ranging 222–242)	Non-parametric test	0.09
Birth weight (g)	1715 (1050–1955)	1900 (1415–2155)	Non-parametric test	0.101
Delivery mode (CS/VD)	1/10	1/10	Chi square test	1
Gender (male/female)	4/7	4/7	Chi square test	1
Feeding (premature infant formula/mixed feeding/breastfeeding)	3/3/5	3/3/5	Chi square test	1
Duration of antibiotics use (d)	10 (9–16)	6 (3–7)	Non-parametric test	0
Prenatal antibiotic (d)	0 (0–3)	0 (0–4)	Non-parametric test	0.949
Postnatal antibiotic (d)	10 (7–13)	4 (3–7)	Non-parametric test	0
PROM (yes/no)	5/6	5/6	Chi square test	0
Histological chorioamnionitis (positive/negative/no. of inspection)	3/5/3	2/5/4	Chi square test	NA

*VD* vaginally delivered, *CS* cesarean section, *PROM* premature rupture of membranes

**Fig. 5:**
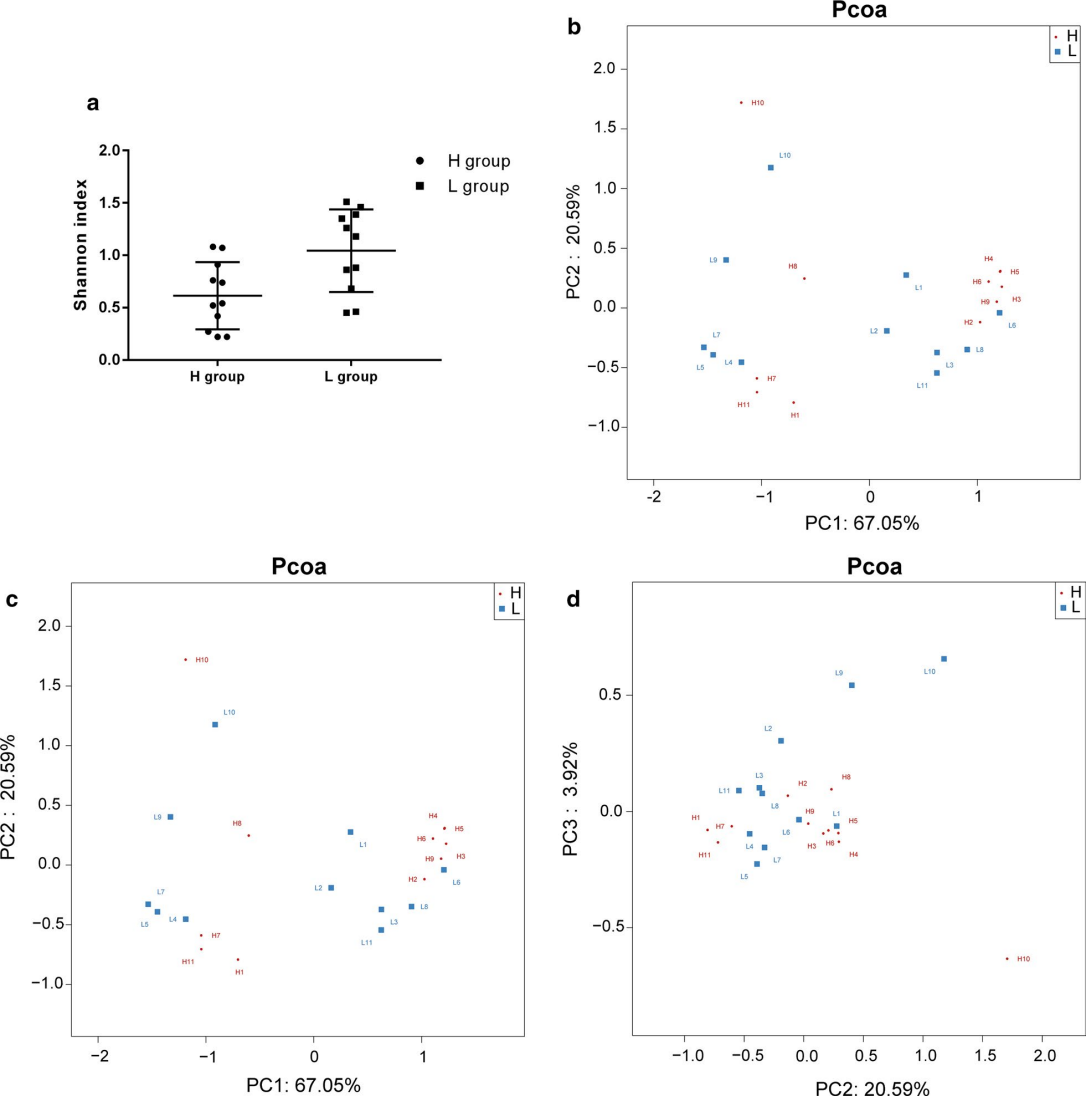
Comparison of the gut microbial communities in the H and L groups. **a** The Shannon index showed no significant difference between the two groups on d14 (*p* > 0.05). PCoA was generated with OTUs (at 97% similarity) present in the different fecal samples. **b** PC1 vs. PC2. **c** PC1 vs. PC3. **d** PC2 vs. PC3

The cut-off was set to 400 sequences per sample and this was the level whereby the rarefaction curves of all samples reached a plateau, indicating that the sequencing depth was sufficient and able to cover most types of bacteria. The Shannon index of the two groups was not significantly different on d14 (*p *> 0.05; Fig. 5a). In PCoA, the bacteria community composition of the H group was not similar to that of L group, as six members (H2-6, 9) gathered to form a subgroup of H, which was well separated from L (Fig. 5b–d).

**Fig. 6:**
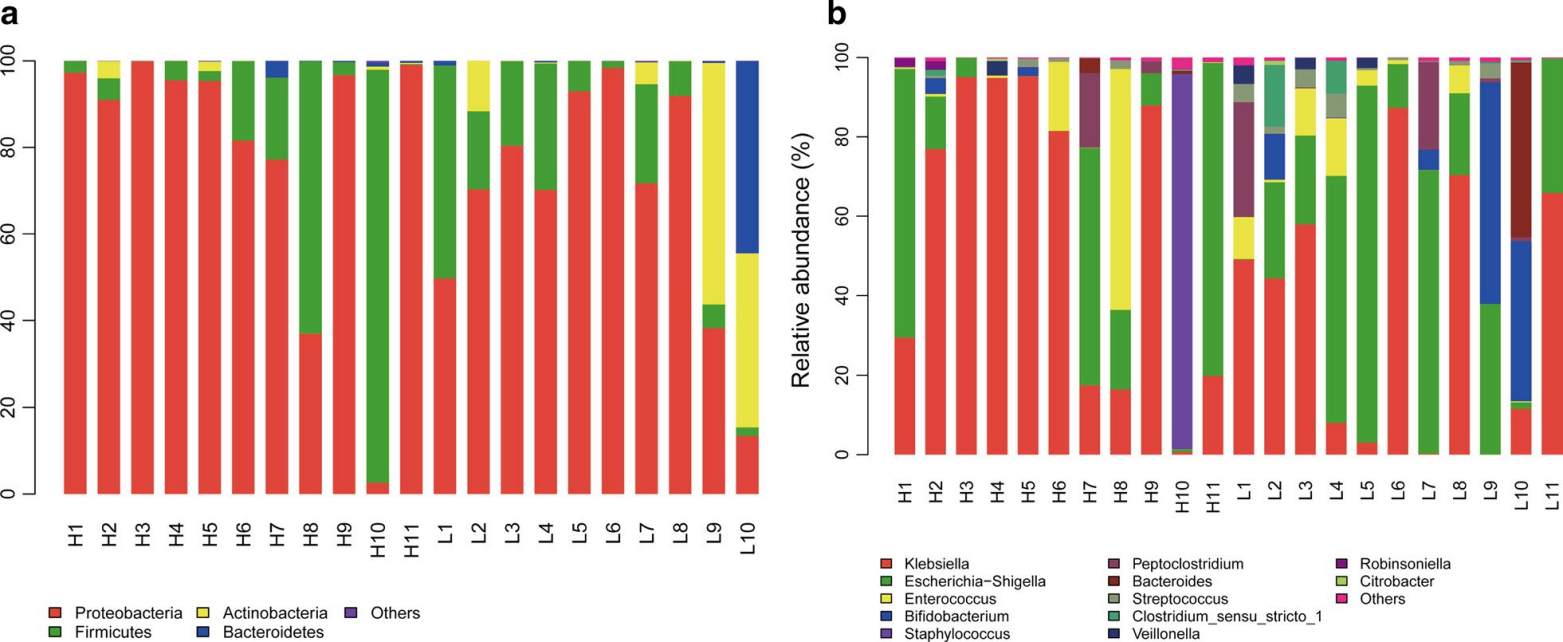
Relative abundance of different microbial groups in guts of preterm infants undergoing antibiotic prophylaxis at high/low doses. **a** At the phylum level. **b** At the genus level. On d14 gut microbiota were generally dominated by the phyla Proteobacteria (79.35% in the H group vs. 70.66% in the L group) and Firmicutes (19.33% in the H group vs. 14.81% in the L group). At the genus level, on d14 the microbiota structure in both groups was similar. The gut microbiota of the two groups remained predominantly populated with members of *Klebsiella* (55.91% in the H group vs. 36.15% in the L group) and *Enterococcus* (23% in the H group vs. 34.22% in the L group). *Shigella* and *Streptococcus* had a tendency to decrease in the H group

**Fig. 7:**
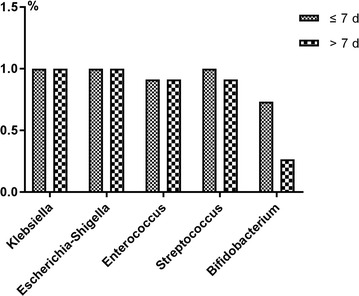
Colonization percentage of five gut bacteria in the H group (> 7d) and L group (≤ 7d). The colonization of Bifidobacterium decreased more significantly in the H group than in the L group (5.47% in the H group vs. 10.24% in the L group, *p *< 0.05)

The relative abundance of dominant bacterial phyla and genera was as follows. On d14 the most abundant phylum was *Proteobacteria* (79.35% in group H and 70.66% in group L; Fig. 6a), followed by *Firmicutes* (19.33% in group H and 14.81% in group L). At the genus level, on d14 the microbiota structure was similar between the two groups. The most abundant genus was *Klebsiella* (55.91% in group H and 36.15 in group L), followed by *Enterococcus* (23% in group H and 34.22% in group L). Moreover, the number of *Shigella* and *Streptococcus* tended to decrease in the H group (Fig. 6b). The relative abundance of *Bifidobacterium* significantly decreased (5.47% in H group and 10.24% in L group, *p *< 0.05; Fig. 7).

The predominant bacteria of H and L groups were similar to the drug resistant strains in NICU of the same period.

**Fig. 8:**
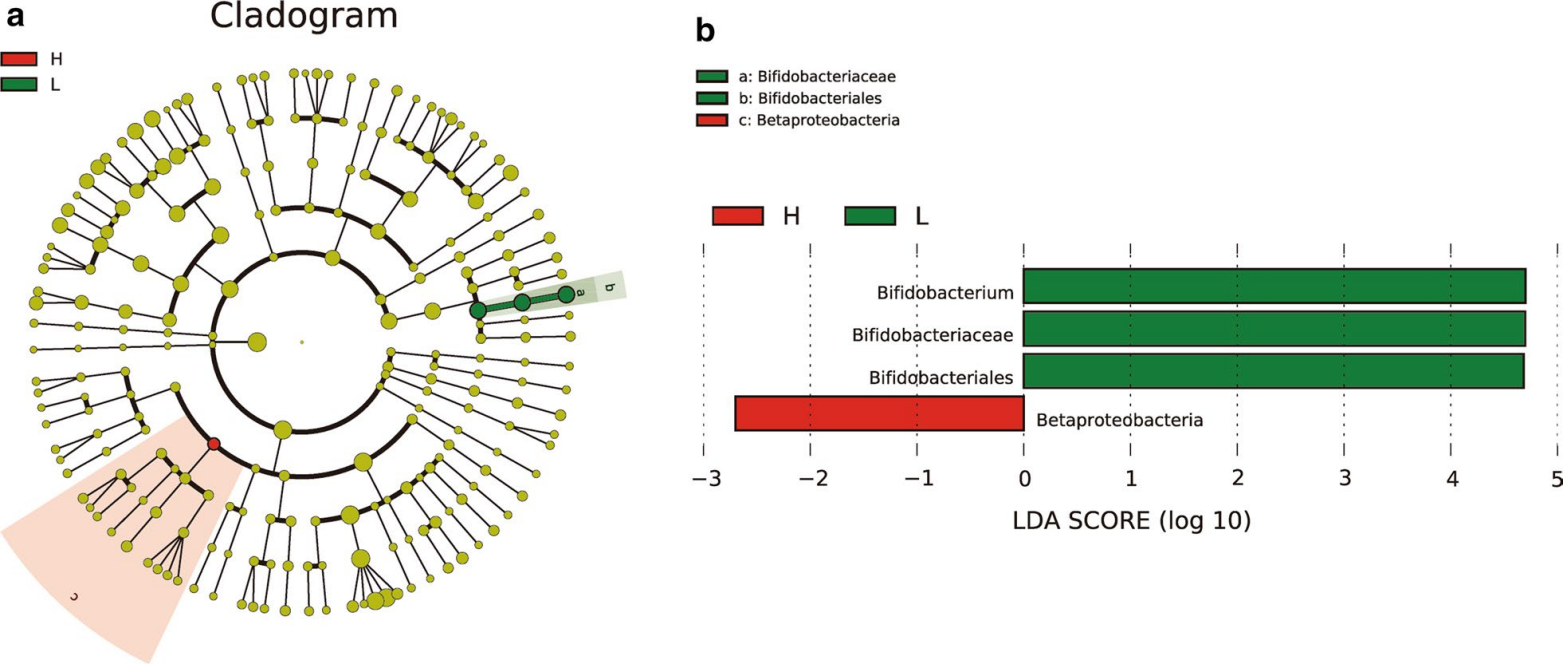
LEfSe analysis showed significant differences in microbial community structure between the H and L groups. **a** Cladogram. **b** Distribution of LDA scores. Betaproteobacteria played an important role in the H group; in the L group, *Bifidobacterium*, Bifidobacteriaceae, and Bifidobacteriales were the prominent microbes

LEfSe analysis of d14 finds the relative abundance of *Betaproteobacteria* was significantly higher in the H group than in the L group; the relative abundance of *Bifidobacterium*, *Bifidobacteriaceae,* and *Bifidobacteriales* was significantly higher in the latter than in the former (Fig. 8).

## Discussion

Antibiotics are of significant importance to modern medicine, and their use has played a pivotal role in the maintenance of human health. Despite this, there are ever-increasing concerns about the negative consequences of antibiotic use, including the collateral damage inflicted on the gut microbiota. Prenatal antibiotic exposure resulted in an increase in *Staphylococcus*, *Streptococcus*, *Serratia*, and *Parabacteroides,* constituting potentially pathogenic communities [15]. Fouhy et al. found that postnatal antibiotic subject exposure (ampicillin and gentamicin within 48 h of birth) resulted in significantly lower levels of *Bifidobacterium* and *Lactobacillus* than in untreated controls 4 weeks after the cessation of treatment [16]. Antibiotics are the most commonly prescribed medications in NICUs. The present study addresses an important knowledge gap, as the effects of prenatal and postnatal antibiotic exposure on preterm infant gut microbiota have not, thus far, been characterized by high throughput sequencing methods that provide sufficient coverage of the entire bacterial community.

In this study, at the phylum level, *Proteobacteria* and *Firmicutes* were predominant in gut microbiota, with *Proteobacteria* more abundant than *Firmicutes*. As there are more harmful genera than beneficial ones in Proteobacteria, an increased prevalence of Proteobacteria is a marker of an unstable microbial community, dysbiosis, and a potential diagnostic criterion of disease [17]. In contrast, there are more beneficial genera than harmful ones within the *Firmicutes* phylum [18]. The increase of *Proteobacteria* and the decrease of *Firmicutes* could increase the incidence of necrotizing enterocolitis (NEC) and infectious diseases [19]. At the genus level, *Klebsiella, Escherichia*-*Shigella* and *Enterococcus* were predominant in gut microbiota, whereas *Bifidobacterium* decreased significantly. It was found that prenatal antibiotic exposure could lead to a reduction in *Bacteroides* and an increase in *Escherichia*-*Shigella* and *Clostridium* in the intestinal microflora of premature infants. It was also found that high intensity antibiotic exposure led to increases in *Beta*-*proteobacteria*. *Bacteroides* and *Bifidobacterium* are intestinal protective bacteria, and a decrease in their population could result in NEC; the opportunistic pathogen *Escherichia*-*Shigella* also results in increased incidence of NEC [19] and increases of *Clostridium* and *Bacteroides* in the intestinal mucosa of NEC cases [20]. It has been found that populations of *Clostridium difficile* increase in NICU environments, which is associated with many intractable infections [21–23]. Therefore, our data implies that preterm infant antibiotic exposure may cause dysbiosis and lead to increased incidences of NEC.

Intriguingly, in both the prenatal antibiotic exposure group and in the high intensity antibiotic exposure group, the colonization of *Bifidobacterium* was delayed and no *Lactobacillus* colonization was observed. *Bifidobacterium* and *Lactobacillus* are very important probiotics. It has been shown that probiotics can restore intestinal micro-ecological balance, repair the intestinal membrane barrier, improve intestinal colonization, and inhibit the excessive growth of opportunistic pathogens [24]. Probiotics help to decrease NEC, late-onset sepsis, and the mortality of preterm infants [25]. It is suggested that the status, the gene phenotype, and the gut microbiota of pregnant women, as well as the delivery mode, could influence the establishment and construction of gut microbiota in newborns [26–29]. Interestingly, microorganisms have also been found in the placenta [30, 31]. Antibiotic treatment for PROM (premature rupture of membranes) pregnant women leads to changes in vaginal microflora, and numbers of *Lactobacillus* have been shown to continuously decline [32]. Further investigations are needed to explore whether or not prenatal antibiotic exposure affects the establishment of gut microbiota in preterm infants by influencing the microbiota in the intestines, vagina, and placenta of pregnant women.

The establishment and development of gut microbiota are influenced by environment factors, gestational age, delivery mode, feeding pattern, and antibiotics [10]. In our study no significant differences were found in the Shannon–Wiener Index and PCoA between the PAT and PAF groups, or between the H and L groups, indicating that antibiotic exposure is not dominant in influencing the establishment and development of gut microbiota at an early stage in premature infants. The NICU has its own unique commensal bacteria and pathogens [33, 34]. In our hospital, antibiotic resistant bacteria colonized in the NICU were mainly *Klebsiella pneumoniae* (40%), *Escherichia coli* (28.57%) and *Acinetobacter baumannii* (20%). The incidence of nosocomial infection was 19.4%. Our data showed that these common resistant bacteria were included in the intestinal microflora at an early stage in premature infants, indicating that bacterial colonization in the NICU may play an important role in the development of gut microbiota in preterm infants. It is widely believed that disturbances in gut microbiota are one of the main causes of NEC and late-onset sepsis [35]. Gut microbiota disturbance often occurs before diseases, and changes in gut microbiota could be considered as an early predictor of preterm infant sepsis [36, 37]. Antibiotic exposure influences the establishment of preterm infant gut microbiota, changing its diversity and richness [15, 38]. Additionally, antibiotic exposure could promote the horizontal gene transfer of antibiotic resistance genes [39]. Whether these effects are synergistic is worth further investigation.

## Conclusions

In summary, prenatal/postnatal antibiotic exposure and the intensity of antibiotic exposure could affect the composition of gut microbiota in preterm infants. It may be safely assumed that environmental factors (e.g. antibiotic-resistant bacteria in NICU) may have a profound impact on the composition of the early gut microbiota in preterm infants with prenatal and postnatal antibiotic exposure. The gut microbiota of preterm infants includes antibiotic-resistant bacteria in NICU, thus posing a threat to the survival of infants and increasing the possibility of therapy failures.

## Additional file

**Additional file 1: Table S1.** Basic information of PAT and PAF groups. **Table S2.** Basic information of H and L groups. **Figure S1.** Rarefaction curve showing the relationship between the sampled sequencing read number and the number of bacterial species that these reads represent. **Figure S2.** Venn diagram showing the numbers of common and unique OTUs among PAT and PAF groups.

## Abbreviations

NICU: neonatal intensive care units
PAT: prenatal antibiotic therapy
PAF: prenatal antibiotic free
OUT: operational taxonomic units
PCoA: principal coordinate analyses
NEC: necrotizing enterocolitis
PROM: premature rupture of membranes
HC: histological chorioamnionitis
VD: vaginally delivered
CS: cesarean section
