# Supplementary material for: Prenatal and postnatal antibiotic exposure influences the gut microbiota of preterm infants in neonatal intensive care units
Source: Ann Clin Microbiol Antimicrob. 2018 Mar 19;17:9. doi: 10.1186/s12941-018-0264-y (PMC5858143; doi:10.1186/s12941-018-0264-y)
Supplement: Supplementary file 1 — Additional file 1: Table S1. Basic information of PAT and PAF groups. Table S2. Basic information of H and L groups. Figure S1. Rarefaction curve showing the relationship between the sampled sequencing read number and the number of bacterial species that these reads represent. Figure S2. Venn diagram showing the numbers of common and unique OTUs among PAT and PAF groups. [file 12941_2018_264_MOESM1_ESM.doc]

**Prenatal and postnatal antibiotic exposure influences the gut microbiota of preterm infants in neonatal intensive care units**

**Additional Figure Legends**

**Figure S1.** Rarefaction curve showing the relationship between the sampled sequencing read number and the number of bacterial species that these reads represent.

**Figure S2.** Venn diagram showing the numbers of common and unique OTUs among PAT and PAF groups.


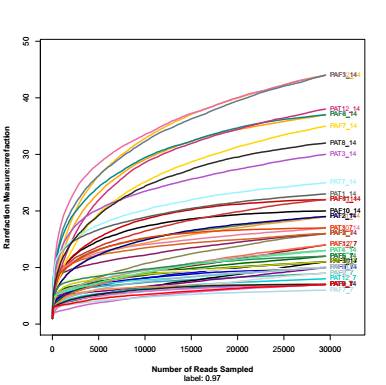


**Figure S1**


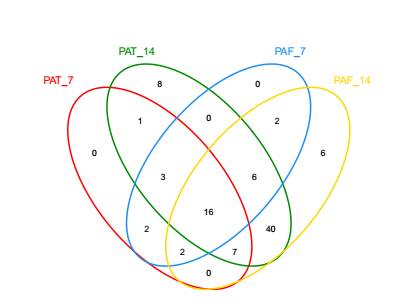


**Figure S2**

**Table S1.** Basic information of PAT and PAF groups

| PAT |  |  |  |  | | |  | |  | |  |  |  |  |
| --- | --- | --- | --- | --- | --- | --- | --- | --- | --- | --- | --- | --- | --- | --- |
| NO. | Sequence No. | Gender | Gestational age (d) | | Delivery mode | Birth weight (g) | | Postnatal antibiotic (d) | | Feeding | | PROM | HC | |
| 320 | PAT-1 | male | 226 | | VD | 2070 | | 3 | | mixed feeding | | yes | negative | |
| 317 | PAT-2 | female | 232 | | VD | 1600 | | 7 | | premature infant formula | | yes | no. of inspection | |
| 314 | PAT-3 | male | 240 | | VD | 2135 | | 3 | | breastfeeding | | yes | negative | |
| 218 | PAT-4 | male | 229 | | CS | 1525 | | 6 | | breastfeeding | | yes | negative | |
| 280 | PAT-5 | male | 223 | | VD | 1500 | | 13 | | breastfeeding | | yes | positive | |
| 414 | PAT-6 | male | 212 | | CS | 1160 | | 5 | | mixed feeding | | yes | no. of inspection | |
| 321 | PAT-7 | male | 226 | | VD | 1880 | | 11 | | mixed feeding | | yes | negative | |
| 372 | PAT-8 | male | 237 | | VD | 1735 | | 7 | | premature infant formula | | yes | no. of inspection | |
| 410 | PAT-9 | male | 225 | | VD | 1715 | | 3 | | breastfeeding | | yes | negative | |
| 216 | PAT-10 | male | 230 | | VD | 2155 | | 3 | | breastfeeding | | yes | positive | |
| 428 | PAT-11 | male | 232 | | VD | 1955 | | 10 | | premature infant formula | | yes | negative | |
| 407 | PAT-12 | male | 219 | | CS | 1895 | | 7 | | premature infant formula | | yes | positive | |
|  |  |  |  | |  |  | |  | |  | |  |  | |
| PAF |  |  |  | |  |  | |  | |  | |  |  | |
| 385 | PAF-1 | female | 225 | | VD | 1730 | | 3 | | mixed feeding | | yes | positive | |
| 327 | PAF-2 | female | 235 | | VD | 1895 | | 7 | | premature infant formula | | yes | negative | |
| 361 | PAF-3 | male | 242 | | VD | 1900 | | 3 | | breastfeeding | | NO | no. of inspection | |
| 386 | PAF-4 | female | 233 | | CS | 1800 | | 8 | | breastfeeding | | yes | negative | |
| 338 | PAF-5 | female | 222 | | VD | 2000 | | 7 | | breastfeeding | | NO | no. of inspection | |
| 250 | PAF-6 | male | 217 | | CS | 1600 | | 5 | | mixed feeding | | yes | positive | |
| 384 | PAF-7 | female | 225 | | VD | 1770 | | 14 | | mixed feeding | | yes | negative | |
| 404 | PAF-8 | female | 236 | | VD | 1950 | | 7 | | premature infant formula | | NO | no. of inspection | |
| 305 | PAF-9 | male | 222 | | VD | 1415 | | 4 | | breastfeeding | | NO | negative | |
| 425 | PAF-10 | female | 226 | | VD | 1955 | | 10 | | breastfeeding | | NO | negative | |
| 326 | PAF-11 | male | 235 | | VD | 1930 | | 9 | | premature infant formula | | yes | negative | |
| 406 | PAF-12 | female | 231 | | CS | 1825 | | 7 | | premature infant formula | | NO | no. of inspection | |

Note:

HC: Histological chorioamnionitis

PROM: premature rupture of membrane

≥ 7d: mixed feeding: premature infant formula and 2d ≤ breastfeeding < 5d; premature infant formula: 7d premature infant formula only and breastfeeding < 2d; breastfeeding: breastfeeding ≥ 5d

≤ 14d: mixed feeding: premature infant formula and 2d ≤ breastfeeding < 10 d; premature infant formula: 14d premature infant formula only and breastfeeding < 2d; breastfeeding: breastfeeding ≥ 10 d

**Table S2.** Basic information of H and L groups

| No. | Sequence No. | Gender | Gestational age(d) | Delivery mode | Birth weight (g) | Duration of antibiotics use (d) | Prenatal antibiotic (d) | Postnatal antibiotic (d) | Feeding | PPROM | HC |
| --- | --- | --- | --- | --- | --- | --- | --- | --- | --- | --- | --- |
| H |  |  |  |  |  |  |  |  |  |  |  |
| 280-14 | H1 | male | 223 | VD | 1500 | 16 | 3 | 13 | breastfeeding | YES | positive |
| 321-14 | H2 | maie | 226 | VD | 1880 | 14 | 3 | 11 | mixed feeding | yes | negative |
| 410-14 | H3 | male | 225 | VD | 1715 | 14 | 3 | 11 | breastfeeding | yes | negative |
| 231-14 | H4 | male | 222 | VD | 1580 | 12 | 0 | 12 | breastfeeding | NO | no. of inspection |
| 425-14 | H5 | female | 226 | CS | 1955 | 10 | 0 | 10 | breastfeeding | NO | negative |
| 243-14 | H6 | female | 227 | VD | 1585 | 10 | 0 | 10 | mixed feeding | yes | no. of inspection |
| 428-14 | H7 | male | 232 | VD | 1955 | 11 | 1 | 10 | premature infant formula | yes | negative |
| 326-14 | H8 | male | 235 | VD | 1930 | 9 | 0 | 9 | premature infant formula | yes | negative |
| 407-14 | H9 | male | 219 | VD | 1895 | 11 | 2 | 9 | premature infant formula | yes | positive |
| 358-14 | H10 | female | 204 | VD | 1050 | 8 | 0 | 8 | mixed feeding | NO | positive |
| 201-14 | H11 | female | 200 | VD | 1125 | 10 | 0 | 10 | breastfeeding | NO | no. of inspection |
|  |  |  |  |  |  |  |  |  |  |  |  |
| L |  |  |  |  |  |  |  |  |  |  |  |
| 385-14 | L1 | female | 225 | VD | 1730 | 3 | 0 | 3 | mixed feeding | yes | positive |
| 320-14 | L2 | male | 226 | VD | 2070 | 6 | 3 | 3 | mixed feeding | yes | negative |
| 216-14 | L3 | male | 230 | VD | 2155 | 7 | 4 | 3 | breastfeeding | yes | positive |
| 314-14 | L4 | male | 240 | VD | 2135 | 5 | 2 | 3 | breastfeeding | yes | negative |
| 218-14 | L5 | male | 229 | CS | 1525 | 7 | 1 | 6 | breastfeeding | yes | negative |
| 305-14 | L6 | male | 222 | VD | 1415 | 4 | 0 | 4 | breastfeeding | NO | negative |
| 372-14 | L7 | male | 237 | VD | 1735 | 7 | 1 | 6 | premature infant formula | yes | no. of inspection |
| 327-14 | L8 | female | 235 | VD | 1895 | 7 | 0 | 7 | premature infant formula | yes | negative |
| 361-14 | L9 | male | 242 | VD | 1900 | 3 | 0 | 3 | breastfeeding | NO | no. of inspection |
| 404-14 | L10 | female | 236 | VD | 1950 | 6 | 0 | 6 | premature infant formula | NO | no. of inspection |
| 338-14 | L11 | female | 222 | VD | 2000 | 6 | 0 | 6 | breastfeeding | NO | no. of inspection |

Note:

HC: Histological chorioamnionitis

PROM: premature rupture of membrane

≥ 7d: mixed feeding: premature infant formula and 2d ≤ breastfeeding < 5d; premature infant formula: 7d premature infant formula only and breastfeeding < 2d; breastfeeding: breastfeeding ≥ 5d

≤ 14d: mixed feeding: premature infant formula and 2d ≤ breastfeeding < 10 d; premature infant formula: 14d premature infant formula only and breastfeeding < 2d; breastfeeding: breastfeeding ≥ 10 d
